# Vitamin D Supplementation in Primary Hyperparathyroidism: Analysis of Benefits and Hazards

**DOI:** 10.3390/nu18152504

**Published:** 2026-08-03

**Authors:** Thomas Audet, Marie-Josée Bégin, Jean-Hugues Brossard, Louis-Georges Ste-Marie, Louis-Philippe Laurin, Catherine Adam, Thi Hoang Lan Nguyen, Marie-Eve Dupuis

**Affiliations:** 1 Division of Endocrinology, Department of Medicine, Centre Hospitalier de l’Université de Montréal, Montréal, QC H2X 0C1, Canada; thomas.audet@umontreal.ca (T.A.); marie-josee.begin@umontreal.ca (M.-J.B.);; 2 Department of Medicine, Université de Montréal, Montréal, QC H3T 1J4, Canadathi.hoang.lan.nguyen@umontreal.ca (T.H.L.N.); 3 Centre de Recherche du Centre Hospitalier de l’Université de Montréal, Montréal, QC H2X 0A9, Canada; 4 Division of Nephrology, Hôpital Maisonneuve-Rosemont, Montréal, QC H1T 2M4, Canada; 5 Centre de Recherche de l’Hôpital Maisonneuve-Rosemont, Montréal, QC H1T 2M4, Canada; 6 Division of Endocrinology, Hôpital Maisonneuve-Rosemont, Montréal, QC H1T 2M4, Canada

**Keywords:** vitamin D supplementation, vitamin D deficiency, primary hyperparathyroidism, hypercalcemia, hungry-bone syndrome, narrative review

## Abstract

Primary hyperparathyroidism (PHPT) and vitamin D deficiency (VD) are two frequent medical conditions in the Western world. Purely by dint of their prevalence, these conditions are commonly comorbid. Moreover, they can interact with one another: VD may stimulate PTH secretion, while PHPT may lead to unregulated vitamin D activation from 25-hydroxyvitamin D (25OHD) to 1,25-dihydroxyvitamin D (1,25OH2D), thereby lowering 25OHD levels and further exacerbating VD. Thus, a real conundrum arises: should the careful clinician replete VD in PHPT or not? On the one hand, both conditions could lead to bone mineral density (BMD) loss if untreated, ultimately resulting in osteoporosis and fragility fractures; on the other hand, vitamin D supplementation could lead to further worsening of hypercalcemia and its dreaded complications. To complicate matters, patients undergoing parathyroidectomy (PTX) after long-standing PHPT can also suffer from postoperative hungry-bone syndrome and symptomatic hypocalcemia, which may be further exacerbated by VD. This narrative review aims to summarize the relevant pathophysiological mechanisms underlying these two diseases and their complex interactions. The latest evidence regarding thresholds and targets for vitamin D supplementation in PHPT will be discussed, with a focus on the balance between benefits and risks.

## 1. Introduction

Primary hyperparathyroidism (PHPT) is a relatively common medical condition around the world, being the most common etiology of hypercalcemia in the outpatient population [1]. Historically defined as hypercalcemia and elevated or non-suppressed parathyroid hormone (PTH) levels, it is nowadays accepted to diagnose PHPT in normocalcemic patients with elevated PTH levels and hypercalciuria [2,3]. Its age-adjusted prevalence in the healthy population of the United States of America has been estimated at 233 and 85 per 100,000 women and men, respectively [1]. In Canada, the prevalence of PHPT amongst community-dwelling adults is estimated at 3.3% and 1.4% (normocalcemic and classical forms, respectively) [4]. Incidence has remained relatively stable at 21.6 per 100,000 person-years in the last decades, after peaking at 82.5 per 100,000 person-years in the 1970s, most likely due to serum calcium being tested more frequently [5]. The consequences of PHPT are varied, ranging from hypercalciuria to nephrolithiasis, chronic kidney disease (CKD), constipation, osteoporosis, neuropsychiatric symptoms and bone/joint pain. Among these, depression and bone/joint pain may not be linked to the level of serum calcium but may still be relieved by definitive surgical cure [6,7]. It has even been suggested to screen every patient for occult urolithiasis given its high prevalence (21%) [3,8]. PHPT and its detrimental effects on bone health have long been known, particularly with respect to cortical bone such as the distal radius, hence the relatively high prevalence of osteoporosis in PHPT cohorts, with some estimates being as high as 48.4% [9,10]. Indeed, the Canadian Multicentre Osteoporosis Study (CaMoS) has shown that patients with PHPT have lower BMD values compared to healthy controls at the lumbar spine, femoral neck and total hip [4].

Historically linked to deficient sunlight exposure with a North-to-South gradient, vitamin D deficiency (VD) is common in Northern resource-rich countries. With better physician awareness in the last few years, vitamin D supplementation has been more widespread, with a decrease in VD prevalence and a reversal of the North-to-South gradient [11]. Population-wide surveys in Canada from 2016 to 2019 estimated that 6.8% of the population aged three to 79 years old has VD, while 19.3% of the population has levels meeting the insufficient range [12].

Purely by chance, one could expect these two conditions to be comorbid in any individual given their respective prevalence, but there seems to be an epidemiological link, for it has been reported that between 36.4 and 81% of patients with PHPT have VD [13,14]. Physiopathological interactions between vitamin D and PHPT have been demonstrated, a topic this review aims to further explore. First, we will begin by explaining the relevant physiology. Then, we will discuss whether it is prudent to replenish VD in patients with PHPT, given the available data on safety and possible benefits, with a specific emphasis on bone mineral density (BMD).

## 2. Overview of Calcium Physiology

**Figure 1:**
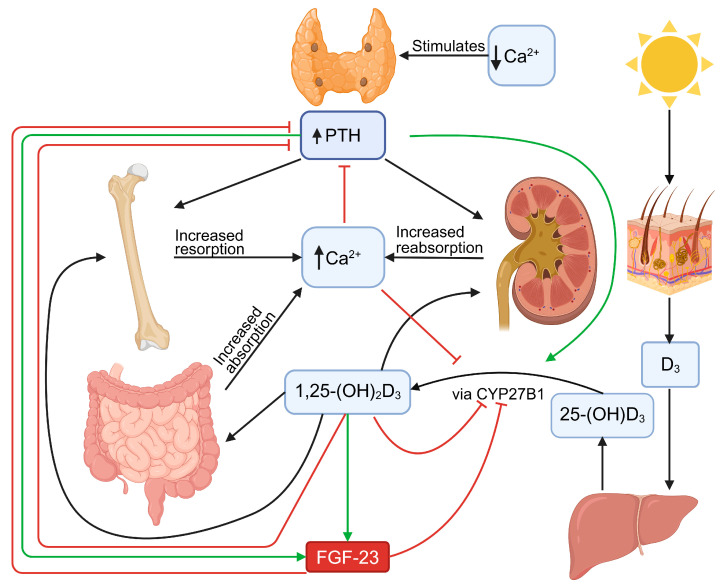
Homeostasis of calcium regulation. Decreased (↓) extracellular calcium acts on the parathyroid gland to increase (↑) PTH secretion. PTH increases calcium reabsorption from the kidneys; increases bone resorption; and increases expression of CYP27B1 in the kidneys. The increase in 1,25-(OH)_2_D3 then serves to increase calcium absorption from the gut, increase bone resorption and increase calcium reabsorption in the kidneys. Positive feedback is shown by green arrowheads, while negative feedback is shown in red. Created in BioRender. Bégin, M. (https://BioRender.com/imjrngj) is licensed under CC BY 4.0.

Before delving into the abnormal pathophysiology of vitamin D metabolism in PHPT, let us briefly review classic calcium homeostasis (Figure 1).

Extracellular ionized calcium is sensed through the Calcium Sensing Receptor (CaSR) [15]. A reduction in calcemia results in PTH secretion and production in the chief cells of the parathyroid glands, which will have three main effects: (1) increased reabsorption of calcium and reduced reabsorption of phosphate in the nephron; (2) increased bone resorption to liberate both calcium and phosphate; and (3) increased expression of CYP27B1 in the kidneys (also known as 1-α-hydroxylase) to convert 25-hydroxyvitamin D (25OHD) into its active form: 1,25-dihydroxyvitamin D (1,25OH2D) [16]. CYP27B1 is also directly stimulated by hypocalcemia and hypophosphatemia. Vitamin D (cholecalciferol) is synthesized from 7-dehydrocholesterol via skin exposure to UVB light. Afterwards, cholecalciferol is constitutively hydroxylated in the liver by CYP2R1 into 25OHD [16]. This step is substrate-dependant and unregulated. Thus, 25OHD levels reflect vitamin D status. This pro-hormone is then hydroxylated via CYP27B1, mainly in the kidneys, into 1,25OH2D, which is considered the active hormone [16]. 1,25OH2D serves to: (1) increase calcium absorption in the gut; (2) increase calcium resorption from bones; and (3) increase calcium reabsorption in the nephron [16]. Both 1,25OH2D and PTH increase the production of fibroblast growth factor 23 (FGF-23) by osteocytes, an important step in feedback, although the effect of this hormone on PTH is still incompletely understood [17].

Methods for measuring vitamin D are not readily available, whereas 25OHD can be measured easily. In the serum, vitamin D metabolites are bound to vitamin D-binding protein (DBP), which maintains their stability and creates an available pool in circulation. Only 1% of the circulating 25OHD and 1,25OH2D is unbound to DBP (the free fraction) [16].

Serum calcium levels are tightly regulated by constant adjustment of PTH secretion, given PTH’s short half-life of a few minutes [18]. Major inhibitory factors of PTH secretion and production are a rise in: (1) serum calcium; (2) 1,25OH2D; and (3) FGF-23 [16]. For its part, CYP27B1 is downregulated by 1,25OH2D itself (creating a feedback loop), as well as a rise in calcemia or in FGF-23 [16]. Vitamin D metabolites are further metabolized via hydroxylation by CYP24A1 (24-α-hydroxylase), a regulated process. The effect of CYP24A1 is enhanced by 1,25OH2D and FGF-23 and decreased by PTH [16]. CYP24A1 competes for the substrate of CYP27B1 (25OHD) and also catabolizes 1,25OH2D [16].

## 3. Search Strategy

A comprehensive search strategy was used to identify relevant articles in multiple electronic databases. PubMed and ClinicalTrials.gov were searched from inception to 02.09.2025. Embase, the Cochrane Library and Scopus were searched for publications from the last 10 years to increase our yield of the most recent literature. Various keywords and Boolean-operator combinations were used to maximize yield. Language was limited to English and/or French. Reviews and systematic reviews were screened for relevant articles (snowball effect). We did not restrict inclusion to interventional data; we also included observational studies and case reports/series to further maximize our yield. Keywords and their derivatives included: “primary hyperparathyroidism”, “vitamin D deficiency”, “vitamin D”, “hypercalcemia”, “nephrocalcinosis” and “nephrolithiasis”.

## 4. Physiopathology of VD in the Context of PHPT

Most patients with PHPT have a single parathyroid adenoma (85% of cases) [19]. Usually, these adenomas present a higher set-point of calcium sensing related to decreased expression of CaSR and an increased mass of parathyroid tissue, maintaining PTH secretion despite the presence of hypercalcemia. Given the increased level of PTH and its effect on CYP27B1, 1,25OH2D levels are usually slightly elevated, whereas 25OHD levels may be low. Thus, a clinical challenge arises: is the low level of 25OHD in PHPT an appropriate homeostatic adaptation to high PTH and 1,25OH2D; or a real state of hypovitaminosis D induced by excessive metabolism of 25OHD? The former hypothesis would suggest no clinical consequence of a low 25OHD level, with surgical cure of PHPT restoring the physiological 25OHD levels to normal; while the latter would suggest a combined detrimental effect of VD and PHPT on many organs, including bone. To better resolve this, let us report the relevant physiopathology of PHPT.

Although thresholds may vary, a recent consensus defined 25OHD serum level in the general population of <30 nmol/L as deficient and levels between 30 to 50 nmol/L as insufficient [20]. An extensive body of literature has studied free and total 25OHD in PHPT. It has been shown that while DBP and total 25OHD are lower in patients with PHPT compared to controls, free 25OHD levels are identical [21,22,23,24,25,26]. The mechanism by which DBP is low in PHPT is not yet understood [27,28]. Thus, low total 25OHD in the serum could be secondary to low DBP, while its free and bioactive fraction remains normal, which resolves post-parathyroidectomy (PTX), supporting the hypothesis of an appropriate adaptation [23,26]. Uncertainty remains regarding these measurements, for two studies did not show any differences in 25OHD, whether free or total, even when accounting for DBP polymorphisms [29,30]. Studies looking at 1,25OH2D in PHPT have shown an increased level of 1,25OH2D, with an increased activation ratio (the ratio of 1,25OH2D to 25OHD), when compared to controls [22,31,32]. This suggests that low 25OHD levels are indeed the result of increased metabolism into 1,25OH2D. Two other studies have reported normal levels of 1,25OH2D in PHPT [33,34].

Further supporting the claim that VD in PHPT is indeed a true form of hypovitaminosis D is the study by Kabadi et al. conducted in 2020, in which, even after PTX, 25OHD levels remained low in a subgroup of patients [32]. These patients had lower calcemia, 25OHD, and 1,25OH2D levels at baseline, with biochemistry profiles suggesting secondary hyperparathyroidism post-PTX, which resolved after 25OHD supplementation [32]. From this data emerges a relevant distinction: though it may be true that PTX can restore 25OHD levels (as shown earlier), in some patients, there is VD concurrent with PHPT, which remains post-PTX. Such VD can be explained by increased metabolism of 25OHD into 1,25OH2D, as previously shown, though it has also been shown that PHPT reduces the serum half-life of 25OHD via increased elimination in feces by the liver [35]. Unfortunately, neither the specific enzyme nor the metabolites involved were described in this study [35]. The role of CYP24A1 in such catabolism in still incompletely understood. A study on 24,25-dihydroxyvitamin D reported normal-low levels (near the lower end of the normal range) of this metabolite in PHPT, and the mechanism by which this occurs remains to be fully elucidated [36].

Further research may yet help identify interactions with other hormones. Levels of FGF-23 are increased in PHPT compared to normal patients [37,38,39]. This has been suggested to be an adaptative mechanism to counteract the PTH-mediated increase in 1,25OH2D; however, conflicting data have been reported [38,39,40]. Furthermore, PTX does not seem to lower FGF-23 levels, though this is debated [37,38,39,40]. The impact of FGF-23 in PHPT is still actively being investigated.

In conclusion, in addition to classical risk factors for hypovitaminosis D, the higher prevalence of VD in PHPT compared to healthy controls may be explained by higher hydroxylation of 25OHD into 1,25OH2D with an increased activation ratio, increased catabolism, and potentially less DBP. When combined, in the absence of a sufficient vitamin D dietary intake or sunlight exposure to replete whole-body stores, these phenomena will result in VD [41].

## 5. Benefits and Safety of VD Treatment in PHPT Regarding Serum and Urinary Calcium

Having outlined the relevant pathophysiology in PHPT, one must now assess whether vitamin D supplementation in the context of PHPT is harmful and/or beneficial. The modern trend regarding physician awareness of VD and more liberal supplementation has also been noticed in PHPT. When comparing vitamin D supplementation in PHPT in the same hospital over time, the percentage of patients receiving any dose of supplemental vitamin D was found to have shifted from 0% in 1984–1991 to 64% in 2010–2014 [42]. The median dose in this study was 800 international units (IU) per day, which reduced the number of patients with VD from 46% to 19%. Neither calcemia nor 24-h urinary calcium levels were different between the two cohorts [42].

There have been 14 observational studies, four published randomized controlled trials (RCTs), one withdrawn RCT, one non-completed RCT, and four systematic reviews with meta-analyses to date on this subject [29,43,44,45,46,47,48,49,50,51,52,53,54,55,56,57,58,59,60,61,62,63,64,65]. All four meta-analyses had different inclusion criteria and strategies, and all four concluded that VD correction in PHPT is safe. It must be noted that only one was registered on PROSPERO and none specifically used the PRISMA guidelines [66,67]. Of the published RCTs, one investigated the effect of 24,25-dihydroxyvitamin D, and another investigated the effect of activated vitamin D in PHPT, neither of which will be further discussed in this review [61,63]. The withdrawn and non-completed RCTs will not be analyzed either [64,65].

The meta-analysis conducted by Ye et al. in 2022 was limited to RCTs and included only two studies on vitamin D vs. placebo [43]. One of these studies was conducted in a non-VD population and randomized patients to active vitamin D (1α-hydroxyvitamin D3) or placebo to assess whether this molecule could be of use in the medical management of PHPT [62]. The other study, by Rolighed et al., which is more relevant to our question, is the only RCT evaluating VD correction in PHPT with supplemental vitamin D [47]. They randomized 46 patients with PHPT and VD, defined as <80 nmol/L, to either 2800 IU of daily D3 or placebo for 1 year, with PTX done at 6 months (mid-study) [47]. The patients had to be at least 18 years old and eligible for PTX to be included. Multiple exclusion criteria were imposed, such as ionized serum calcium at entry >1.6 mmol/L, serum creatinine >120 μmol/L, pregnancy, use of cinacalcet or alfacalcidiol, and many more. The most frequent surgical criterion for patients was prevalent osteoporosis (n = 20/46). Calcemia under vitamin D supplementation did not significantly change in either group before PTX, from a mean baseline of 1.40 mmol/L (95% CI [confidence interval] 1.37; 1.43) in the intervention group and 1.41 mmol/L in the placebo group (95% CI 1.38; 1.44) [47]. Supplemental vitamin D was able to raise 25OHD from 50.2 nmol/L to 94.2 nmol/L at week 25 in the intervention group. This had a non-significant effect on 24-h calciuria according to the authors. Baseline 24-h calciuria was 9.7 mmol/d (95% CI 7.4; 11.9) and 9.2 (95% CI 7.2; 11.1) mmol/d in the intervention group and the placebo group, respectively [47]. No induced hypercalcemia requiring exclusion from the study happened (as pre-specified in the safety measures of the protocol), with the highest ionized calcemia reached at 1.65 mmol/L. Two follow-up studies with respect to the main one were completed [68,69]. No additional effects of supplemental vitamin D were found regarding well-being, quality of life, multiple muscular indices, or health-related quality of life [68,69].

The three remaining meta-analyses included non-randomized studies as well as RCTs. None have the same inclusion criteria, nor have the most recent ones reused the same search strategies as the previous ones. Thus, some of the older studies on VD correction in PHPT have been excluded from the most recent meta-analysis [45]. The earliest of these, published in 2014 by Shah et al., had a restrictive search strategy with few keywords, though they allowed multiple formulations of vitamin D supplements and included international meeting abstracts [46]. Thus, two of the included studies were not peer-reviewed works at the time, although they were later published in 2014 and 2018, and accounted for 12.5–30.1% of the weight of the total population [46,58,60]. Here, VD was defined as 25OHD < 50 nmol/L. Overall, the meta-analysis by Shah et al. showed that VD correction in PHPT was efficacious with respect to repleting 25OHD stores, while calcemia did not increase during the study period.

The second meta-analysis, conducted by Loh et al. in 2019, again used a language-restricted search strategy, with few keywords, and was limited to native vitamin D or D3 (although they did include studies that used D2) [44]. There was no clear definition of VD. A striking feature is that it did not include the only RCT published on VD and PHPT, which was published in its inclusion period [47]. Again, it showed the effectiveness of repleting VD and the innocuity regarding calcemia.

The latest meta-analysis was conducted by Song et al. in 2021 [45]. Its search strategy was rigorous, and it included trials regardless of the vitamin D formulation used. Vitamin D deficiency was defined as 25OHD levels <75 nmol/L. It included 11 of the 13 available observational studies available at the time [55,60]. They also identified two RCTs but could not perform a meta-analysis on these, for some data could not be retrieved [47,61]. Furthermore, one of the RCTs evaluated the effect of 24,25-dihydroxyvitamin D in the medical management of PHPT, which is a completely different aspect [61]. The meta-analysis by Song et al. concluded that vitamin D supplementation in PHPT is efficacious and safe. Supplemental vitamin D was able to raise total 25OHD levels by 55.2 nmol/L (95% CI 37.5; 72.9), with considerable heterogeneity (*p* < 0.00001, *I*^2^ = 98%) [45,70]. The mean difference in calcemia with supplemental vitamin D was −0.01mmol/L (95% CI −0.04; 0.01) with substantial heterogeneity (*p* = 0.03, *I*^2^ = 51%) [70]. Meanwhile, the mean difference in PTH levels was −1.70 pmol/L (95% CI −3.05; −0.34) with substantial heterogeneity (*p* = 0.0003, *I*^2^ = 69%) [45,70]. They could only evaluate two studies reporting on calciuria and its change under VD repletion [52,54]. When pooled, no significant change in 24-h urine calcium was found, though the confidence interval was wide. The estimated change was 0.912 mmol/day (95% CI −0.93; 2.76) with substantial heterogeneity (*p* = 0.12, *I*^2^ = 58%) [45,70]. Unfortunately, they did not analyze the data from the study by Grey et al. in this calculation, which was included in the systematic review [50]. Furthermore, they only focused on calciuria and did not include safety data regarding urolithiasis reported in the different studies [56,57,58,59].

Of the 14 observational studies identified in this review, one cohort trial was not included in any of the meta-analyses [29]. It was mainly a biochemical study looking at DBP and free versus total vitamin D levels between PHPT patients and healthy controls [29]. In the PHPT arm, 56 patients received 14,000 IU of vitamin D3, weekly, for 12 weeks. The authors showed the innocuity of vitamin D, with a non-significant decline in calcemia from 2.82 ± 0.22 mmol/L to 2.79 ± 0.22 mmol/L (*p =* 0.10) at 12 weeks, with no reported adverse effects [29].

In conclusion, the available studies demonstrate that replenishing vitamin D levels in PHPT is efficacious in correcting VD and is safe regarding calcemia, with a tendency to lower it. This data applies to mild PHPT, with most studies excluding patients with calcemia >2.9–3.0 mmol/L.

## 6. Impact of VD and Its Repletion on Bone Health in PHPT

A relationship between VD, the severity of PHPT, and osteoporosis has been hinted at by many for decades. Already in 1987, Patron was publishing data about overt bone disease with low vitamin D and higher PTH levels [71]. More recent data has linked higher 1,25OH2D in PHPT to lower BMD, a sign of a more active disease [31,72]. A protective effect of higher 25OHD levels on lumbar BMD has been suggested by some, yet this is still debated [34,73,74,75]. Additional unstudied or unknown factors remain, such as vitamin D receptor genotype [72,76]. This matter is further complicated by observational studies reporting no differences in BMD or osteoporotic fractures when stratifying PHPT patients by vitamin D levels [14,72,77,78]. Two studies even suggest that, although BMD does not correlate with 25OHD levels, trabecular bone score (TBS [an index of bone microarchitecture]) may be a better index to assess bone health and microarchitecture in PHPT [79,80].

Bone histomorphometric analysis in PHPT and VD may explain the impacts of VD on bone structure. In one study comparing groups with levels of 25OHD of 30 nmol/L ± 7.5 vs. 80 nmol/L ± 27.5, patients in the former group had an increased extent of osteoid surface (OS/BS) of 31% ± 10 vs. 21% ± 7 (*p* < 0.03) when compared with the latter group [34]. The percentage of bone surface with tetracycline labels (mineralizing surface [MS/BS]) was also greater in the former group, at 30% ± 11, vs. the latter group, at 14% ± 9 (*p* = 0.05) [34]. In another study, 25OHD levels < 20 nmol/L, when compared to >20 nmol/L, were correlated with lower cortical width (Ct.Wi), increased cancellous bone volume (BV/TV), increased trabecular number (Tb.N) and lower trabecular separation (Tb.Sp) [81]. In both studies, low levels of 25OHD were correlated with higher PTH levels. Altogether, these histomorphometric data suggest that VD in PHPT induces increased bone remodeling due to higher levels of PTH rather than a VD profile (i.e., osteomalacia). This latest disease can coexist with a PHPT bone disease profile (i.e., osteitis fibrosa) but remains quite rare [82,83]. This also suggests that PTH, in the context of VD and PHPT, has an anabolic effect on trabecular bone (lumbar spine) and a catabolic effect on cortical bone (radius and total hip) [81]. However, one study did not replicate these observations, though it included few patients with VD [84]. This last group was notably younger, heavier, and more likely to be male: all of which could have confounded the results of the study.

To further assess the impact of VD on bone health in PHPT, let us examine the data when repleting VD in PHPT. Of the 15 aforementioned original studies on VD repletion in PHPT (14 observational studies and one RCT), only four report data on BMD [47,49,50,55]. Unfortunately, direct comparison of BMD results between the studies is impossible, for different dual-energy X-ray absorptiometry (DXA) scans with different coefficients of variation were used. To account for this, one could use T-score variation after VD repletion to compare results across studies, but only two studies report such data [49,55]. Only one study specifically reports on the absence of new clinical fractures at 17 ± 3 months under vitamin D repletion, though there was no systematic surveillance with imaging [56]. Notably, 15 out of the 28 patients (54%) in this study were also prevalent bisphosphonate users. Table 1 summarizes the data.

Three studies report data on the lumbar spine. The study by Kantorovich et al. reported a significant improvement of 6.3% mean annual gain in BMD when comparing BMD at 13 ± 8 months [49]. It must be noted that VD repletion was accomplished within a short period of time (5 weeks) at the start of the trial, and no further vitamin D was given to the patients. The other two reported no change or a non-significant improvement in BMD [47,50]. In the RCT conducted by Rolighed et al., it must be noted that while the 1.0% gain (95% CI −0.5; 2.5) in lumbar spine BMD was not significant in the group under vitamin D at 6 months, there was a statistically (*p* < 0.05) and clinically significant difference between these patients and the placebo group, in which there was a −1.5% decrease (95% CI −2.7; −0.2) [47].

Only one study reports on total hip BMD [47]. At six months, there was a non-significant gain of 0.2% (95% CI −0.9; 1.3) in total hip BMD [47]. This result is non-statistically different from the placebo group, however, for the latter had a significant gain of 0.7% (95% CI −0.8; 2.1) at 6 months [47].

Femoral neck BMD is subject to much more technical variation and should therefore be compared with caution [85]. However, this is the site with the most data. The study by Kantorovich et al. is the only one to report a significant improvement in BMD of 8.2% mean annual gain when comparing BMD at 13 ± 8 months [49]. The study by Velayoudom-Cephise et al. only reported on T-scores [55]. After six months of vitamin D repletion, a statistically significant improvement in the mean T-score at the femoral neck from −2.23 ± 0.49 to −1.75 ± 0.86 (*p* = 0.001) was observed [55]. Two other studies reported stable BMD [47,50].

One last area of interest in regard to PHPT is the distal forearm, specifically the proximal ⅓, for it is a site rich in cortical bone [3]. The study by Rolighed et al. is the only one to report such data, with non-significant changes in both groups at six months [47].

In conclusion, VD, in the setting of PHPT, seems to be linked to a greater effect of PTH on bone metabolism rather than a primary VD profile bone histomorphometry. Repleting VD has no deleterious effect on BMD and may have a protective effect on lumbar BMD at six months.

## 7. Analysis of the Available Data with an Emphasis on Bone Health

To properly analyze the 15 original studies done on VD repletion in PHPT, one must keep in mind that there is no unique definition of either VD or insufficiency among the studies. Up to seven of them do not clearly define a threshold for VD: three of these are biochemical studies, and another three study derivatives of vitamin D as a potential treatment for PHPT [29,48,49,54,61,62,63]. Most of the studies (six) define VD as <50 nmol/L, although three define it as <75 nmol/L, with the rest using varying thresholds. It must be noted that thresholds regarding VD are highly debated in the literature, and most papers do not specifically address the issue if a different threshold in PHPT exists. A complete rundown of this debate is beyond the scope of this review but let us remind the reader of the two major theories.

Arguing for >50 nmol/L as adequate for bone health are two major organizations claiming that most cases of nutritional rickets happen with a serum 25OHD level <34 nmol/L [20,41]. As 25OHD rises, PTH reaches a plateau at around 50 nmol/L of 25OHD [86,87,88]. Furthermore, most of the population may experience a beneficial effect of reaching 50 nmol/L [41]. These last statements are highly controversial for experts arguing for >75 nmol/L, for other studies demonstrate that PTH reaches a plateau at 70–80 nmol/L [89,90,91]. Fracture prevention data from meta-analyses on vitamin D supplementation only suggest a reduction in hip fractures at >60 nmol/L, with most trials reaching around 75 nmol/L, and some even reporting fracture reduction at 100 nmol/L of 25OHD [92,93]. However, it must be noted that none of the meta-analyses on fracture risk reduction with vitamin D included patients with PHPT. Only one of the studies on VD repletion in PHPT included data on the absence of fractures at 17 ± 3 months with vitamin D supplementation (with a 25OHD increase from 32.2 ± 1.7 nmol/L to 136.4 ± 11.6 nmol/L) [56]. Therefore, one must be cautious when extrapolating fracture prevention data with vitamin D supplementation from the general osteoporosis population and applying it to PHPT. Unfortunately, no studies have looked at the impact of VD repletion on bone histomorphometry in PHPT. Histomorphometric cross-sectional studies have demonstrated a more aggressive bone disease related to PHPT rather than a VD profile (i.e., osteomalacia) in patients with PHPT and VD when compared to PHPT alone [34,81]. Thus, treating PHPT should be the main focus to limit its impact on bone health, and meta-analyses do not show a meaningful impact of vitamin D on PTH levels in PHPT [45]. One interesting avenue for improving BMD would be PTX, if patients meet the criteria, for BMD gains are indeed prevalent. Supplemental 25OHD in the perioperative period does not improve BMD gains further [94]. The only data supporting vitamin D supplementation in PHPT regarding bone health relate to the protective effect of vitamin D on lumbar BMD [47,49]. While the well-designed study by Rolighed et al. did not show a significant gain in lumbar BMD, it did show a statistically and clinically significant protective effect versus placebo against BMD loss [47]. The data regarding the femoral neck is promising yet of greater heterogeneity and therefore cannot be as easily relied upon.

To better apply this debate to PHPT, one must also consider safety issues with regard to vitamin D supplementation, such as induced or increased hypercalcemia and hypercalciuria in patients already dealing with these issues at baseline. In the 15 original studies reported in this review, out of 531 patients, only six significant hypercalcemic events were recorded [52,53,57]. Of the ten studies reporting on calciuria, seven report on data after VD repletion [47,49,50,51,52,54,55,56,57,60]. When combined, out of 246 patients, 20 instances of incident hypercalciuria happened (which were not always well defined), with no incident urolithiasis [50,52,60]. Of the studies that did not report on calciuria, only two mentioned the absence of incident urolithiasis [58,59]. A recently published review specifically addressing the risk of urolithiasis with vitamin D supplementation in PHPT has revealed more insights into this complicated relationship [95]. While vitamin D supplementation may increase the risk of urolithiasis, VD may too [95]. Other lithogenic factors related to individual patient characteristics are also involved. The absence of systematic renal imaging and thorough pre/post-intervention assessments in the included studies limited their ability to draw a conclusion [95]. From the available studies, the risk of urolithiasis or hypercalciuria does not seem to be greatly increased in the short term.

Furthermore, some studies loosely established the definition of PHPT in their respective cohorts, and about half did not report on calciuria. Thus, familial hypocalciuric hypercalcemia (FHH) cannot be excluded. The reader should note that although calciuria has traditionally been used as a marker to differentiate PHPT from FHH, it must be noted that this index may be skewed in profound VD (i.e., under 25 nmol/L), with a lower fractional excretion of calcium being reported, falsely leading to an inappropriate diagnosis of FHH [96].

While most studies used different inclusion criteria, most were limited to non-surgical patients, with mild or asymptomatic PHPT. Mild PHPT was usually defined as non-severe hypercalcemia (<2.9–3.0 mmol/L), with calciuria <10 mmol/d when it was measured. Notable exceptions were studies in which PTX was part of the protocol, though it must be noted that PTX was performed only after a very short time window [47,51]. Exclusion criteria were plentiful, from CKD to comorbidities affecting calcium metabolism such as sarcoidosis, again limiting the external validity of the results from these studies to real-life patients. Some even excluded patients after an adverse event occurred, thereby adding some nuance to the safety of vitamin D supplementation in PHPT regarding calcemia.

Finally, this review cannot claim to apply to normocalcemic PHPT, for only two studies included these patients, with very few patients overall [49,59]. Also, it has not addressed the issue of preventing hungry-bone syndrome post-PTX with preoperative vitamin D, though the latest systematic review on this matter does not support its use [97]. A more recent RCT addressing this topic has also failed to demonstrate any impact of repleting 25OHD levels pre-PTX, even when aiming for >75 nmol/L [98].

## 8. Concluding Thoughts

A clear and evidence-based threshold for vitamin D in PHPT has not yet been established. Recent guidelines have acknowledged that although a definitive threshold cannot be established, based on available data, it seems prudent to recommend levels of 25OHD >75 nmol/L, but this is purely based on expert opinion [3]. Others, with a more cautious approach, argue for a threshold between 50 to 75 nmol/L [99,100]. A detailed report published alongside the 2022 guidelines acknowledges that repletion with vitamin D, while safe, should be applied cautiously to prevent worsening of serum and urinary calcium [101]. Of the 15 original studies on vitamin D repletion and PHPT, most of them ended up achieving a target serum 25OHD between 75 and 100 nmol/L. The majority of these studies used daily doses of vitamin D supplementation ranging between 1000 and 2800 IU for one to six months (excluding outliers, for some protocols were quite aggressive, with doses of vitamin D of up to 50,000 IU twice weekly) [49]. As discussed earlier, this resulted in very few adverse events. Thus, in well-selected patients with mild asymptomatic PHPT and VD, a careful clinician could safely carry out supplementation, with the goal of reaching >75 nmol/L, knowing that very few adverse events would occur, and a protective effect on lumbar BMD could be expected. A well-designed RCT specifically addressing the topic of 25OHD repletion with different targets and different protocols would greatly improve the scientific literature on this topic.

## Figures and Tables

**Table 1:** Summary of the data on vitamin D’s effects on BMD from the available studies ^a^.

Study	Vitamin D Posology and Duration of Therapy	BMD Assessment (DXA)	Parameter	Lumbar Spine	Femoral Neck	Total Hip	Proximal ⅓ of Distal Forearm
Kantorovich et al. [49] N = 5	D2 50,000 IU twice weekly 5 weeks	Lunar T0 and 13 ± 8 months	BMD	0.965 ± 0.237 → 1.029 ± 0.274 ^b^	0.618 ± 0.101 → 0.663 ± 0.061 ^b^	N/A	N/A
			T-score	−1.35 ± 0.79 → −0.72 ± 0.86	−2.96 ± 0.38 → −2.47 ± 0.2	N/A	N/A
Grey et al. [50] N = 21	D3 50,000 IU weekly × 4 weeks then monthly 12 months	Lunar T0 and 12 months	BMD	1.02 ± 0.16 → 1.02 ± 0.05	0.78 ± 0.18 → 0.79 ± 0.07	N/A	N/A
Velayoudom-Cephise et al. [55] N = 22	D2 800–1200 IU daily 3–6 months	Unknown T0 and 6 months	T-score	N/A	−2.23 ± 0.49 → −1.75 ± 0.86	N/A	N/A
Rolighed et al. [47] N = 40	D3 2800 IU daily 6 months	Hologic T0 and week 25	BMD	0.89 (0.84–0.94) → 0.90 (0.85–0.95) ^c^	0.67 (0.62–0.72) → 0.67 (0.63–0.72)	0.80 (0.75–0.86) → 0.81 (0.75–0.86)	0.59 (0.53–0.65) → 0.58 (0.53–0.64)
	Placebo 6 months		BMD	0.93 (0.86–1.01) → 0.92 (0.85–0.99) ^b,c^	0.75 (0.69–0.82) → 0.75 (0.68–0.81)	0.89 (0.81–0.96) → 0.89 (0.82–0.97)	0.59 (0.52–0.66) → 0.58 (0.52–0.65)

^a^ BMD is reported as g/cm^2^. Values are presented as means with their standard deviations or ranges. N/A = not applicable (some studies have not reported data on all BMD sites). IU = international units. ^b^ denotes a change above the least-significant change. ^c^ denotes a statistically significant change between the placebo and vitamin D groups (*p* < 0.05).

## Data Availability

No new data were created or analyzed in this study. Data sharing is not applicable to this article.

## Abbreviations

The following abbreviations are used in this manuscript:

25OHD	25-hydroxyvitamin D, 25(OH)D
1,25OH2D	1,25-dihydroxyvitamin D; 1,25(OH)_2_D
BMD	Bone mineral density
CI	Confidence interval
CKD	Chronic kidney disease
DBP	Vitamin D-binding protein
DXA	Dual-energy X-ray absorptiometry
FGF23	Fibroblast growth factor 23
FHH	Familial hypocalciuric hypercalcemia
IU	International units
MS/BS	Mineralizing surface
OS/BS	Extent of osteoid surface
PHPT	Primary hyperparathyroidism
PTH	Parathyroid hormone
PTX	Parathyroidectomy
RCT	Randomized controlled study
TBS	Trabecular bone score
VD	Vitamin D deficiency

## References

[1] Yeh M.W., Ituarte P.H., Zhou H.C., Nishimoto S., Liu I.L., Harari A., Haigh P.I., Adams A.L. (2013). Incidence and prevalence of primary hyperparathyroidism in a racially mixed population. J. Clin. Endocrinol. Metab..

[2] Consensus Development Task Force on Diagnosis and Management of Asymptomatic Primary Hyperparathyroidism (2003). Asymptomatic primary hyperparathyroidism: Standards and guidelines for diagnosis and management in Canada: Consensus Development Task Force on Diagnosis and Management of Asymptomatic Primary Hyperparathyroidism position paper. Endocr. Pract..

[3] Bilezikian J.P., Khan A.A., Silverberg S.J., Fuleihan G.E., Marcocci C., Minisola S., Perrier N., Sitges-Serra A., Thakker R.V., Guyatt G. (2022). Evaluation and Management of Primary Hyperparathyroidism: Summary Statement and Guidelines from the Fifth International Workshop. J. Bone Miner. Res..

[4] Berger C., Almohareb O., Langsetmo L., Hanley D.A., Kovacs C.S., Josse R.G., Adachi J.D., Prior J.C., Towheed T., Davison K.S. (2015). Characteristics of hyperparathyroid states in the Canadian multicentre osteoporosis study (CaMos) and relationship to skeletal markers. Clin. Endocrinol..

[5] Wermers R.A., Khosla S., Atkinson E.J., Achenbach S.J., Oberg A.L., Grant C.S., Melton L.J. (2006). Incidence of Primary Hyperparathyroidism in Rochester, Minnesota, 1993-2001: An Update on the Changing Epidemiology of the Disease. J. Bone Miner. Res..

[6] Bargren A.E., Repplinger D., Chen H., Sippel R.S. (2011). Can biochemical abnormalities predict symptomatology in patients with primary hyperparathyroidism?. J. Am. Coll. Surg..

[7] Benge J.F., Perrier N.D., Massman P.J., Meyers C.A., Kayl A.E., Wefel J.S. (2009). Cognitive and affective sequelae of primary hyperparathyroidism and early response to parathyroidectomy. J. Int. Neuropsychol. Soc..

[8] Tay Y.D., Liu M., Bandeira L., Bucovsky M., Lee J.A., Silverberg S.J., Walker M.D. (2018). Occult urolithiasis in asymptomatic primary hyperparathyroidism. Endocr. Res..

[9] Chappard C., Roux C., Laugier P., Paillard M., Houillier P. (2006). Bone status in primary hyperparathyroidism assessed by regional bone mineral density from the whole body scan and QUS imaging at calcaneus. Jt. Bone Spine.

[10] Reid L.J., Muthukrishnan B., Patel D., Seckl J.R., Gibb F.W. (2019). Predictors of Nephrolithiasis, Osteoporosis, and Mortality in Primary Hyperparathyroidism. J. Clin. Endocrinol. Metab..

[11] Oskarsson V., Eliasson M., Salomaa V., Reinikainen J., Männistö S., Palmieri L., Donfrancesco C., Sans S., Costanzo S., de Gaetano G. (2022). Influence of geographical latitude on vitamin D status: Cross-sectional results from the BiomarCaRE consortium. Br. J. Nutr..

[12] Canada S. (2023). Table: 13-10-0872-01 Vitamin D Status of Canadians, Combined Cycles, by Age Group and Sex. https://www150.statcan.gc.ca/t1/tbl1/en/tv.action?pid=1310087201.

[13] Tassone F., Gianotti L., Baffoni C., Visconti G., Pellegrino M., Cassibba S., Croce C.G., Magro G., Cesario F., Attanasio R. (2013). Vitamin D status in primary hyperparathyroidism: A Southern European perspective. Clin. Endocrinol..

[14] Moosgaard B., Vestergaard P., Heickendorff L., Melsen F., Christiansen P., Mosekilde L. (2005). Vitamin D status, seasonal variations, parathyroid adenoma weight and bone mineral density in primary hyperparathyroidism. Clin. Endocrinol..

[15] Murray S.L., Wolf M. (2024). Calcium and Phosphate Disorders: Core Curriculum 2024. Am. J. Kidney Dis..

[16] Bilezikian J.P., The American Society for Bone and Mineral Research (2025). Primer on the Metabolic Bone Diseases and Disorders of Mineral Metabolism.

[17] Agoro R., White K.E. (2023). Regulation of FGF23 production and phosphate metabolism by bone–kidney interactions. Nat. Rev. Nephrol..

[18] Khan M., Jose A., Sharma S. (2025). Physiology, Parathyroid Hormone. StatPearls.

[19] Stack B.C., Singer M.C., Shonka D.C. (2025). Medical and Surgical Treatment of Parathyroid Diseases: An Evidence-Based Approach.

[20] Munns C.F., Shaw N., Kiely M., Specker B.L., Thacher T.D., Ozono K., Michigami T., Tiosano D., Mughal M.Z., Mäkitie O. (2016). Global Consensus Recommendations on Prevention and Management of Nutritional Rickets. J. Clin. Endocrinol. Metab..

[21] Meng L., Su C., Shapses S.A., Al-Dayyeni A., He Y., Wang X. (2020). Lower total 25-hydroxyvitamin D but no difference in calculated or measured free 25-hydroxyvitamin D serum levels in patients with primary hyperparathyroidism. J. Steroid Biochem. Mol. Biol..

[22] Meng L., Su C., Shapses S.A., Wang X. (2022). Total and free vitamin D metabolites in patients with primary hyperparathyroidism. J. Endocrinol. Investig..

[23] Meng L., Shapses S.A., Wang X. (2025). Parathyroidectomy Reduces Inflammatory Cytokines and Increases Vitamin D Metabolites in Patients With Primary Hyperparathyroidism. Endocr. Pract..

[24] Wang X., Shapses S.A., Wei S., Sukumar D., Ghosh J. (2013). Vitamin D-binding protein levels in female patients with primary hyperparathyroidism. Endocr. Pract..

[25] Wang X., Shapses S.A., Al-Hraishawi H. (2017). Free and Bioavailable 25-Hydroxyvitamin D Levels in Patients with Primary Hyperparathyroidism. Endocr. Pract..

[26] Wang X., Sheng Z., Meng L., Su C., Trooskin S., Shapses S.A. (2019). 25-Hydroxyvitamin D and Vitamin D Binding Protein Levels in Patients With Primary Hyperparathyroidism Before and After Parathyroidectomy. Front. Endocrinol..

[27] Jassil N.K., Sharma A., Bikle D., Wang X. (2017). Vitamin D Binding Protein and 25-Hydroxyvitamin D Levels: Emerging Clinical Applications. Endocr. Pract..

[28] Bouillon R., Schuit F., Antonio L., Rastinejad F. (2019). Vitamin D Binding Protein: A Historic Overview. Front. Endocrinol..

[29] Dos Santos L.M., Ohe M.N., Pallone S.G., Nacaguma I.O., Kunii I.S., da Silva R.E.C., Maeda S.S., Vieira J.G.H., Lazaretti-Castro M. (2023). Levels of bioavailable, and free forms of 25(OH)D after supplementation with vitamin D(3) in primary hyperparathyroidism. Endocrine.

[30] Battista C., Guarnieri V., Carnevale V., Baorda F., Pileri M., Garrubba M., Salcuni A.S., Chiodini I., Minisola S., Romagnoli E. (2017). Vitamin D status in primary hyperparathyroidism: Effect of genetic background. Endocrine.

[31] Moosgaard B., Vestergaard P., Heickendorff L., Mosekilde L. (2007). Plasma 1,25-dihydroxyvitamin D levels in primary hyperparathyroidism depend on sex, body mass index, plasma phosphate and renal function. Clin. Endocrinol..

[32] Kabadi U.M. (2020). Low 25-Hydroxyvitamin D in Primary Hyperparathyroidism: Enhanced Conversion Into 1,25-Hydroxyvitamin D May Not Be “True” Deficiency. JBMR Plus.

[33] Nordenström E., Westerdahl J., Lindergård B., Lindblom P., Bergenfelz A. (2002). Multifactorial risk profile for bone fractures in primary hyperparathyroidism. World J. Surg..

[34] Silverberg S.J., Shane E., Dempster D.W., Bilezikian J.P. (1999). The effects of vitamin D insufficiency in patients with primary hyperparathyroidism. Am. J. Med..

[35] Clements M.R., Davies M., Fraser D.R., Lumb G.A., Mawer E.B., Adams P.H. (1987). Metabolic inactivation of vitamin D is enhanced in primary hyperparathyroidism. Clin. Sci..

[36] Nikkilä M.T., Saaristo J.J. (1989). Serum vitamin D metabolite concentrations in primary hyperparathyroidism. Ann. Med..

[37] Columbu C., Rendina D., Gennari L., Pugliese F., Carnevale V., Salcuni A.S., Chiodini I., Battista C., Tabacco P., Guarnieri V. (2025). Phosphate metabolism in primary hyperparathyroidism: A real-life long-term study. Endocrine.

[38] Yamashita H., Yamashita T., Miyamoto M., Shigematsu T., Kazama J.J., Shimada T., Yamazaki Y., Fukumoto S., Fukagaw M., Noguchi S. (2004). Fibroblast growth factor (FGF)-23 in patients with primary hyperparathyroidism. Eur. J. Endocrinol..

[39] Witteveen J.E., van Lierop A.H., Papapoulos S.E., Hamdy N.A. (2012). Increased circulating levels of FGF23: An adaptive response in primary hyperparathyroidism?. Eur. J. Endocrinol..

[40] Hassani S., Afkhamizadeh M., Teimouri A., Najaf Najafi M., Vazifeh Mostaan L., Mohebbi M. (2020). Evaluation of Serum Level of FGF23 and 1,25(OH)(2)D(3) in Primary Hyperparathyroidism Patients Before and After Parathyroidectomy. Int. J. Gen. Med..

[41] Ross A.C., Taylor C.L., Yaktine A.L., Del Valle H.B., Institute of Medicine Committee to Review Dietary Reference Intakes for Vitamin D and Calcium (2011). The National Academies Collection: Reports funded by National Institutes of Health. Dietary Reference Intakes for Calcium and Vitamin D.

[42] Walker M.D., Cong E., Lee J.A., Kepley A., Zhang C., McMahon D.J., Bilezikian J.P., Silverberg S.J. (2015). Low vitamin D levels have become less common in primary hyperparathyroidism. Osteoporos. Int..

[43] Ye Z., Silverberg S.J., Sreekanta A., Tong K., Wang Y., Chang Y., Zhang M., Guyatt G., Tangamornsuksun W., Zhang Y. (2022). The Efficacy and Safety of Medical and Surgical Therapy in Patients with Primary Hyperparathyroidism: A Systematic Review and Meta-Analysis of Randomized Controlled Trials. J. Bone Miner. Res..

[44] Loh H.H., Lim L.L., Yee A., Loh H.S., Vethakkan S.R. (2019). Effect of vitamin D replacement in primary hyperparathyroidism with concurrent vitamin D deficiency: A systematic review and meta-analysis. Minerva Endocrinol..

[45] Song A., Zhao H., Yang Y., Liu S., Nie M., Wang O., Xing X. (2021). Safety and efficacy of common vitamin D supplementation in primary hyperparathyroidism and coexistent vitamin D deficiency and insufficiency: A systematic review and meta-analysis. J. Endocrinol. Investig..

[46] Shah V.N., Shah C.S., Bhadada S.K., Rao D.S. (2014). Effect of 25 (OH) D replacements in patients with primary hyperparathyroidism (PHPT) and coexistent vitamin D deficiency on serum 25(OH) D, calcium and PTH levels: A meta-analysis and review of literature. Clin. Endocrinol..

[47] Rolighed L., Rejnmark L., Sikjaer T., Heickendorff L., Vestergaard P., Mosekilde L., Christiansen P. (2014). Vitamin D treatment in primary hyperparathyroidism: A randomized placebo controlled trial. J. Clin. Endocrinol. Metab..

[48] LoCascio V., Adami S., Galvanini G., Ferrari M., Cominacini L., Tartarotti D. (1985). Substrate-product relation of 1-hydroxylase activity in primary hyperparathyroidism. N. Engl. J. Med..

[49] Kantorovich V., Gacad M.A., Seeger L.L., Adams J.S. (2000). Bone mineral density increases with vitamin D repletion in patients with coexistent vitamin D insufficiency and primary hyperparathyroidism. J. Clin. Endocrinol. Metab..

[50] Grey A., Lucas J., Horne A., Gamble G., Davidson J.S., Reid I.R. (2005). Vitamin D repletion in patients with primary hyperparathyroidism and coexistent vitamin D insufficiency. J. Clin. Endocrinol. Metab..

[51] Grubbs E.G., Rafeeq S., Jimenez C., Feng L., Lee J.E., Evans D.B., Perrier N.D. (2008). Preoperative vitamin D replacement therapy in primary hyperparathyroidism: Safe and beneficial?. Surgery.

[52] Isidro M.L., Ruano B.n. (2009). Biochemical effects of calcifediol supplementation in mild, asymptomatic, hyperparathyroidism with concomitant vitamin D deficiency. Endocrine.

[53] Tucci J.R. (2009). Vitamin D therapy in patients with primary hyperparathyroidism and hypovitaminosis D. Eur. J. Endocrinol..

[54] Bevilacqua M., Invernizzi M., Righini V., Carda S., Cisari C. (2011). Different vitamin D substrate-product relationship after oral vitamin D supplementation in familial benign hypercalcemia, primary hyperparathyroidism, and healthy controls. Eur. J. Endocrinol..

[55] Velayoudom-Cephise F.L., Foucan L., Soudan B., Cardot-Bauters C., Vantyghem M.C., D’Herbomez M., Tison-Muchery F., Wemeau J.L. (2011). Half of the patients with primary hyperparathyroidisms have a vitamin D deficiency: Aggravating the osseous attack. Presse Med..

[56] Rao R.R., Randeva H.S., Sankaranarayanan S., Narashima M., Möhlig M., Mehanna H., Weickert M.O. (2012). Prolonged treatment with vitamin D in postmenopausal women with primary hyperparathyroidism. Endocr. Connect..

[57] Wagner D., Xia Y., Hou R. (2013). Safety of vitamin D replacement in patients with primary hyperparathyroidism and concomitant vitamin D deficiency. Endocr. Pract..

[58] Rathi M.S., Gonzalez S., Wright D., Ellis N.R., Peacey S.R. (2014). Management of hypovitaminosis D in patients with primary hyperparathyroidism. J. Endocrinol. Investig..

[59] Das G., Eligar V., Govindan J., Bondugulapati L.N., Okosieme O., Davies S. (2015). Impact of vitamin D replacement in patients with normocalcaemic and hypercalcaemic primary hyperparathyroidism and coexisting vitamin D deficiency. Ann. Clin. Biochem..

[60] Tripto-Shkolnik L., Jaffe A., Liel Y. (2018). The impact of vitamin D status and parameters of calcium metabolism in patients with primary hyperparathyroidism. QJM.

[61] Netelenbos J.C., Asscheman H., Lips P., Van der Vijgh W.J., Jongen M.J., Van Ginkel F., Hackeng W.H. (1986). Absence of effect of 24,25-dihydroxyvitamin D3 in primary hyperparathyroidism. J. Clin. Endocrinol. Metab..

[62] Lind L., Wengle B., Sørensen O.H., Wide L., Akerström G., Ljunghall S. (1989). Treatment with active vitamin D (alphacalcidol) in patients with mild primary hyperparathyroidism. Acta Endocrinol..

[63] Lind L., Wengle B., Lithell H., Ljunghall S. (1991). Reduction in serum alkaline phosphatase levels by treatment with active vitamin D (alphacalcidol) in primary and secondary hyperparathyroidism and in euparathyroid individuals. Scand. J. Urol. Nephrol..

[64] Columbia University Vitamin D Repletion in Primary Hyperparathyroidism. ClinicalTrials.gov Identifier: NCT01306656. Updated [16.04.2019]. NCT01306656.

[65] Columbia University Primary Hyperparathyroidism (PHPT): Early Effect of Vitamin D. ClinicalTrials.gov Identifier: NCT01329666. Updated [05.03.2015]. NCT01329666.

[66] Page M.J., McKenzie J.E., Bossuyt P.M., Boutron I., Hoffmann T.C., Mulrow C.D., Shamseer L., Tetzlaff J.M., Akl E.A., Brennan S.E. (2021). The PRISMA 2020 statement: An updated guideline for reporting systematic reviews. BMJ.

[67] Schiavo J.H. (2019). PROSPERO: An International Register of Systematic Review Protocols. Med. Ref. Serv. Q..

[68] Rolighed L., Rejnmark L., Sikjaer T., Heickendorff L., Vestergaard P., Mosekilde L., Christiansen P. (2015). No beneficial effects of vitamin D supplementation on muscle function or quality of life in primary hyperparathyroidism: Results from a randomized controlled trial. Eur. J. Endocrinol..

[69] Åberg V., Norenstedt S., Zedenius J., Sääf M., Nordenström J., Pernow Y., Nilsson I.L. (2015). Health-related quality of life after successful surgery for primary hyperparathyroidism: No additive effect from vitamin D supplementation: Results of a double-blind randomized study. Eur. J. Endocrinol..

[70] Chandler J., Cumpston M., Higgins J.P.T., Li T., Page M.J., Thomas J., Welch V.A., Cochrane C. (2019). Cochrane Handbook for Systematic Reviews of Interventions.

[71] Patron P., Gardin J.P., Paillard M. (1987). Renal mass and reserve of vitamin D: Determinants in primary hyperparathyroidism. Kidney Int..

[72] Christensen M.H., Apalset E.M., Nordbø Y., Varhaug J.E., Mellgren G., Lien E.A. (2013). 1,25-dihydroxyvitamin D and the vitamin D receptor gene polymorphism Apa1 influence bone mineral density in primary hyperparathyroidism. PLoS ONE.

[73] Walker M.D., Cong E., Lee J.A., Kepley A., Zhang C., McMahon D.J., Silverberg S.J. (2015). Vitamin D in Primary Hyperparathyroidism: Effects on Clinical, Biochemical, and Densitometric Presentation. J. Clin. Endocrinol. Metab..

[74] Inoue Y., Kaji H., Hisa I., Tobimatsu T., Naito J., Iu M.F., Sugimoto T., Chihara K. (2008). Vitamin D status affects osteopenia in postmenopausal patients with primary hyperparathyroidism. Endocr. J..

[75] Lee J.H., Kim J.H., Hong A.R., Kim S.W., Shin C.S. (2017). Skeletal effects of vitamin D deficiency among patients with primary hyperparathyroidism. Osteoporos. Int..

[76] Kobayashi T., Sugimoto T., Kobayashi A., Chihara K. (1998). Vitamin D receptor genotype is associated with cortical bone loss in Japanese patients with primary hyperparathyroidism. Endocr. J..

[77] Şengül Ayçiçek G., Aydoğan B., Şahin M., Emral R., Erdoğan M.F., Güllü S., Başkal N., Çorapçıoğlu D. (2023). The impact of vitamin D deficiency on clinical, biochemical and metabolic parameters in primary hyperparathyroidism. Endocrinol. Diabetes Nutr. (Engl. Ed.).

[78] Viccica G., Cetani F., Vignali E., Miccoli M., Marcocci C. (2017). Impact of vitamin D deficiency on the clinical and biochemical phenotype in women with sporadic primary hyperparathyroidism. Endocrine.

[79] Carnevale V., Manfredi G., Romagnoli E., De Geronimo S., Paglia F., Pepe J., Scillitani A., D’Erasmo E., Minisola S. (2004). Vitamin D status in female patients with primary hyperparathyroidism: Does it play a role in skeletal damage?. Clin. Endocrinol..

[80] Santos L.M., Ohe M., Pallone S., Nacaguma I., Kunii I., Silva R., Brandão C.M., Vieira J.G., Lazaretti-Castro M. (2023). Concentrations of total, bioavailable, and free 25OHD in individuals with and without primary hyperparathyroidism and their correlations to DXA and trabecular bone score. Arch. Endocrinol. Metab..

[81] Stein E.M., Dempster D.W., Udesky J., Zhou H., Bilezikian J.P., Shane E., Silverberg S.J. (2011). Vitamin D deficiency influences histomorphometric features of bone in primary hyperparathyroidism. Bone.

[82] Kaplan F.S., Soffer S.R., Fallon M.D., Haddad J.G., Dalinka M., Raffensperger E.C. (1988). Osteomalacia as a very late manifestation of primary hyperparathyroidism. Clin. Orthop. Relat. Res..

[83] Bordier P., De Seze S., Hioco D., Lichtwitz A., Mazabraud A. (1956). Osteomalacia with biochemical syndrome of hyperparathyroidism or primary hyperparathyroidism with clinical syndrome of osteomalacia. Presse Med. (1893).

[84] Walker M.D., Nishiyama K.K., Zhou B., Cong E., Wang J., Lee J.A., Kepley A., Zhang C., Guo X.E., Silverberg S.J. (2016). Effect of Low Vitamin D on Volumetric Bone Mineral Density, Bone Microarchitecture, and Stiffness in Primary Hyperparathyroidism. J. Clin. Endocrinol. Metab..

[85] Shuhart C., Cheung A., Gill R., Gani L., Goel H., Szalat A. (2024). Executive Summary of the 2023 Adult Position Development Conference of the International Society for Clinical Densitometry: DXA Reporting, Follow-up BMD Testing and Trabecular Bone Score Application and Reporting. J. Clin. Densitom. Off. J. Int. Soc. Clin. Densitom..

[86] Thomas M.K., Lloyd-Jones D.M., Thadhani R.I., Shaw A.C., Deraska D.J., Kitch B.T., Vamvakas E.C., Dick I.M., Prince R.L., Finkelstein J.S. (1998). Hypovitaminosis D in Medical Inpatients. N. Engl. J. Med..

[87] Gong M., Wang K., Sun H., Wang K., Zhou Y., Cong Y., Deng X., Mao Y. (2023). Threshold of 25(OH)D and consequently adjusted parathyroid hormone reference intervals: Data mining for relationship between vitamin D and parathyroid hormone. J. Endocrinol. Investig..

[88] Saliba W., Barnett O., Rennert H.S., Lavi I., Rennert G. (2011). The relationship between serum 25(OH)D and parathyroid hormone levels. Am. J. Med..

[89] Heaney R.P., Holick M.F. (2011). Why the IOM recommendations for vitamin D are deficient. J. Bone Miner. Res..

[90] Lips P. (2006). Vitamin D physiology. Prog. Biophys. Mol. Biol..

[91] Mendes M.M., Hart K.H., Lanham-New S.A., Botelho P.B. (2020). Suppression of Parathyroid Hormone as a Proxy for Optimal Vitamin D Status: Further Analysis of Two Parallel Studies in Opposite Latitudes. Nutrients.

[92] Bischoff-Ferrari H.A., Willett W.C., Wong J.B., Giovannucci E., Dietrich T., Dawson-Hughes B. (2005). Fracture prevention with vitamin D supplementation: A meta-analysis of randomized controlled trials. JAMA.

[93] Bischoff-Ferrari H.A., Willett W.C., Orav E.J., Lips P., Meunier P.J., Lyons R.A., Flicker L., Wark J., Jackson R.D., Cauley J.A. (2012). A pooled analysis of vitamin D dose requirements for fracture prevention. N. Engl. J. Med..

[94] Norenstedt S., Pernow Y., Zedenius J., Nordenström J., Sääf M., Granath F., Nilsson I.-L. (2014). Vitamin D supplementation after parathyroidectomy: Effect on bone mineral density-a randomized double-blind study. J. Bone Miner. Res. Off. J. Am. Soc. Bone Miner. Res..

[95] Castellano E., Saponaro F. (2025). Vitamin D Metabolism and the Risk of Renal Stones: A Focus on PHPT. Metabolites.

[96] Jayasena C.N., Mahmud M., Palazzo F., Donaldson M., Meeran K., Dhillo W.S. (2011). Utility of the urine calcium-to-creatinine ratio to diagnose primary hyperparathyroidism in asymptomatic hypercalcaemic patients with vitamin D deficiency. Ann. Clin. Biochem..

[97] Witteveen J.E., van Thiel S., Romijn J.A., Hamdy N.A.T. (2013). Hungry bone syndrome: Still a challenge in the post-operative management of primary hyperparathyroidism: A systematic review of the literature. Eur. J. Endocrinol..

[98] Salman M.A., Rabiee A., Salman A.A., Youssef A., Shaaban H.E., Ftohy T., Maurice K.K., Balamoun H. (2021). Role of vitamin D supplements in prevention of hungry bone syndrome after successful parathyroidectomy for primary hyperparathyroidism: A prospective study. Scand. J. Surg..

[99] Insogna K.L. (2018). Primary Hyperparathyroidism. N. Engl. J. Med..

[100] Yedla N., Kim H., Sharma A., Wang X. (2023). Vitamin D Deficiency and the Presentation of Primary Hyperparathyroidism: A Mini Review. Int. J. Endocrinol..

[101] Bilezikian J.P., Silverberg S.J., Bandeira F., Cetani F., Chandran M., Cusano N.E., Ebeling P.R., Formenti A.M., Frost M., Gosnell J. (2022). Management of Primary Hyperparathyroidism. J. Bone Miner. Res..

